# Advanced Techniques for Securing Airway in Mucopolysaccharidoses and the Impact of New Therapeutic Approaches

**DOI:** 10.7759/cureus.10582

**Published:** 2020-09-22

**Authors:** Yousef M Mohammed, Safeera Khan

**Affiliations:** 1 Anesthesia and Pain Management, California Institute of Behavioral Neurosciences & Psychology, Fairfield, USA; 2 Medicine, Damascus University, Damascus, SYR; 3 Internal Medicine, California Institute of Behavioral Neurosciences & Psychology, Fairfield, USA

**Keywords:** anesthesia complications, mucopolysaccharidoses, airway techniques, new therapy approaches

## Abstract

Airway management in patients suffering from mucopolysaccharidoses (MPS) is one of the most difficult anesthesiologic challenges. MPS is a group of rare, inherited diseases caused by the absence or malfunctioning of lysosomal enzymes needed to break down macromolecules called glycosaminoglycans (GAGs). MPS is associated with clinical symptoms and physical features, which all together contribute to the high incidence of difficulty in providing airway during surgical procedures. We used PubMed as our main database (PubMed Advanced Search Builder) to search for relevant literature. At first, we looked for the prevalence of MPS worldwide. Then, we searched for airway management complications in the MPS population using the keywords: “Mucopolysaccharidoses,” “Anesthesia complications,” and “airway management.” Another search was carried out to look for new therapeutic agents and explore their impacts on body organs. We reviewed the finalized articles to explore how anesthesiologists used different airway techniques. We discovered that video laryngoscope and I-gel aided fiber-optic intubation have been available in recent years and have been used uneventfully in several patients. We presented recommendations regarding preoperative and intraoperative preparation to avoid airway-related complications in the future. We realized that many therapy approaches had been suggested, especially after further understanding of the pathophysiology of MPS. However, more investigation needs to be conducted to determine their efficacy and explore if there is any impact on airway management.

## Introduction and background

The global prevalence of all types of MPS has been one case in 25,000 births [1]. Data collected from two studies, one conducted in Japan between 1982 and 2009 and other in Switzerland between 1975 and 2008, showed 41 patients with MPS were identified in Switzerland, and 467 patients were identified in Japan making a combined birth prevalence of 1.56 and 1.53 per 100,000 live births, respectively [2]. MPS is a group of inherited metabolic diseases caused by genetic defects in the production of the lysosomal enzymes that degrade glycosaminoglycans (GAGs), previously known as mucopolysaccharidoses (MPS). Accumulation of GAGs in various tissues and organs is associated with a progressive decline in organ function and considerable tissue damage. All MPS are inherited in an autosomal recessive pattern, which affects males and females equally, whereas the only one inherited as an X-recessive disorder is MPS II, which affects only males [3]. Table 1 shows the classification of different MPS diseases and the enzymes deficient in them. 

**Table 1:** Classification of Mucopolysaccharidoses With the Deficient Enzymes and the Accumulated Glycosaminoglycans

Mucopolysaccharidoses Disease	Deficient Enzyme	Accumulated Glycosaminoglycans
Mucopolysaccharidoses I: IH as Hurler syndrome, IH/S as Hurler-Scheie syndrome, and IS as Scheie syndrome	α-L-iduronidase	Dermatan sulfate, Heparan sulfate
Mucopolysaccharidoses II: Hunter syndrome	Iduronate sulfate sulfatase	Dermatan sulfate, Heparan sulfate
Mucopolysaccharidoses III A–D: Sanfilippo syndrome	A: Heparan-S-sulfaminidase, B: N-Acetyl-α-d-glucosaminidase, C: Acetyl-Co-A glucosaminidase, D: N-Acetylglucosidase N-acyltransferase	Heparan sulfate
Mucopolysaccharidoses IV A, B: Morquio syndrome	A: Galactosamine-6-sulfate sulfatase, B: Galactosamine-6-sulfate sulfatase	A: Keratan sulfate, Chondroitin 6-sulfate; B: Keratan sulfate, Chondroitin 6-sulfate
Mucopolysaccharidoses VI: Maroteaux-Lamy syndrome	N-acetyl-galactosamine α-4-sulfate sulfatase	Dermatan sulfate
Mucopolysaccharidoses VII: Sly syndrome	β-glucuronidase	Dermatan sulfate, Heparan sulfate, Chondroitin sulfate
Mucopolysaccharidoses IX	Hyaluronidase 1	Hyaluronan
Mucopolysaccharidoses V and VIII terms are no longer used.		

Patients usually are healthy at birth, and the pathological manifestations like involvement of respiratory, cardiac, and joint systems; organ enlargement; coarsened facial features; skeletal abnormalities; nose and throat problems; and mental retardation occur later in life [3-5]. Various potential problems with MPS patients related to the accumulation of GAGs in the respiratory airways can cause hypertrophy of adenoids, tonsils, and laryngopharynx, which in turn can make anesthetic airway very difficult [6]. MPS patients undergo surgeries for different organ involvement. A study including 527 patients with MPS II showed that 83.7% of patients needed a surgical intervention during disease progression [7]. Umbilical and inguinal hernia repair, myringotomies and related procedures, tracheostomy, nasal and sinus procedures, spinal surgeries, cardiac valve replacement, orthopedic surgery, adenotonsillectomy, abdominal interventions, and oral surgery are usually the most common required surgeries for patients with MPS I and are prevalent in other MPS types too [8,9]. Other risk factors causing a difficult airway access include hypoplastic tracheal cartilages as well as restrictive lung disease and stiff temporomandibular joints, which altogether can make laryngoscopy and endotracheal tube placement very difficult and can even become worse by increased oral and nasal secretions [10]. Also, facemask difficulties have been reported for different reasons such as anatomical issues, oral and nasal secretions, or even mucosal swelling after intubation [11,12]. The purpose of this paper is to review the previous literature and find out why MPS patients represent a big challenge for anesthesiologists. We will try to look at other anesthesiologists’ experiences, investigate the recent techniques, provide a guideline of recommendations regarding airway management, and explore if there is any impact of new suggested MPS treatments on airway obtaining.

## Review

The study aimed to examine the anesthesiologic complications related to airway management in MPS patients. We carried out our search using PubMed as our main database (PubMed Advanced Search Builder). First, we looked for the epidemiology of MPS worldwide, and then we used a combination of the following terms: “Mucopolysaccharidoses,” “Anesthesia complications,” and “airway management”. Another search was conducted specifically to investigate the recent MPS treatments. The search included all ages and was limited to the articles available in the English language and involved the studies on human subjects only. Prospective studies and randomized controlled trials were lacking regarding the evidence base for airway management. Preferred Reporting Items for Systematic Reviews and Meta-Analyses (PRISMA) guidelines were not used in our traditional review. A full-quality appraisal of all the collected studies was not done. We selected our studies after screening the articles by going through the titles and abstracts to find the relevant literature on the topic of interest. Among the selected studies, there were three clinical trials regarding new MPS therapy approaches that have not been published yet. Also, additional studies were added when bibliographic references of identified articles were reviewed to find articles not identified by electronic search. The collection of the articles for this study was done ethically and legally.

Airway management risk factors

**Figure 1:**
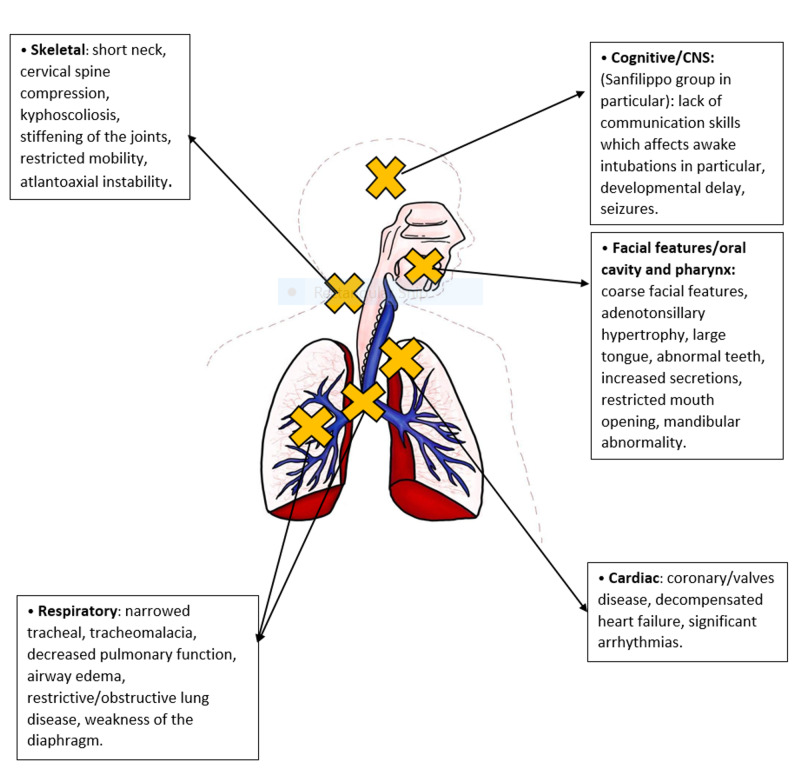
Difficulties in Anesthesia Administration of Mucopolysaccharidoses

MPS diseases are a group of gradually progressive diseases, with a significant influence on the quality of life. Many MPS patients require surgical interventions during disease advancement. MPS is associated with both clinical and physical challenges (Figure 1) that can lead to serious difficulties regarding airway management [3-6,10]. A study showed that while undergoing surgery, nearly 20% of deaths with MPS I patients were directly associated with airway complications [8]. A retrospective study of 31 patients with mixed MPS types evaluated upper and lower airway findings on flexible bronchoscopy while undergoing anesthetic procedures and reported that the most common features were GAGs deposit in the upper airways in 72%, laryngomalacia in 31%, and lower airway deposits in 46% [13]. All these findings can seriously endanger patients’ lives during anesthesia. Figure 1 shows the difficulties encountered during anesthesia administration of MPS patients.

Descriptions of difficult airways in MPS patients 

In the selected case series, we discovered the incidence of difficult airways during the anesthetic procedures in mixed MPS patients from different age groups. Two case series reported difficult face mask placement in 20 out of 141 procedures and 11 out of 44 procedures [11,14]. The overall rate of difficult face mask in two series of different MPS types varies between 6.7% and 33.3% [15,16]. Furthermore, a retrospective analysis of 54 MPS patients showed that intubation was performed in 65 out of 232 anesthetic procedures (28%), added that 19 intubations (29.2%) among those were considered difficult (a Cormack and Lehane score of three or more), and showed that there is a link between older age and increased rates of difficult airways without any association with MPS type [17]. Patients from older age groups would have more anatomical alterations than younger groups. The lack of association between MPS type and airway problems can be due to the inadequate number of patients with specific MPS types. Some studies concluded that higher age is associated only with difficult intubation. In contrast, any difficulty in the placement of either face mask or laryngeal mask airway is not associated with more senior ages [18].

On the other hand, two case series linked difficult intubation to both MPS type and higher age [11,14]. Also, two reviews of a large number of MPS III patients revealed no difficulties with mask ventilation and tracheal intubation [19,20]. The lack of problems regarding mask ventilation and tracheal intubation in MPS III patients means that the level of difficult airways differs among MPS types. It is probably due to the severe somatic involvement in some MPS types and the milder or no involvement in others, particularly MPS III. The overall frequency of difficult endotracheal intubation, including the failed attempts in several series regardless of MPS types, is between 3.17% and 44.2% [11,12,14,19]. The results of the reviewed series are summarized in Table 2.

**Table 2:** Airway Management Results in Mucopolysaccharidoses Case Series

Author Name	Year of Publication	Number of Patients	Number of Anesthetics	Airway Management Results
Frawley et al. [11]	2012	17 mixed types	141	Difficult face mask placement was observed in 14.81% of cases (20 anesthetics). Difficult intubation was seen in 28% of cases (40 procedures).
Walker et al. [12]	1994	34 mixed types	89	Eight patients developed a problem regarding face mask usage (six patients due to anatomical issues and two patients due to excess secretions). Tracheal intubation was conducted on 60 occasions (29 patients); it was difficult on 20 occasions (33.3%). Laryngeal mask airway was used on 12 occasions and was used three times in one patient after a failed intubation.
Moores et al. [14]	1996	28 mixed types	99	Face mask was used in 44 procedures and was difficult to use in 11 procedures (25%). Intubation was conducted 52 times with 23 difficult intubations. Laryngeal mask airway was used in two procedures.
Megens et al. [15]	2014	19 mixed types (including 17 Hurler patients)	136	The use of face mask was found difficult in nine out of 130 cases. Difficult tracheal intubation was found in 24 out of 67 cases, video laryngoscope was successful in 89%, direct laryngoscope was successful in 67%, and fiber-optic scope was uncomplicated in 20%.
Clark BM et al. [16]	2017	18 mixed types	49	Two out of six patients had difficult mask ventilation. Difficult intubation was seen in three out of 36 occasions (8.3%) and was conducted either via fiber-optic intubation or video laryngoscope in 15 procedures. Video laryngoscope was able to secure native airways uneventfully in 12 out of 18 patients. However, it could not keep a glottic view in one patient, so synchronous use of video laryngoscope and fiber-optic intubation was conducted.
Scaravilli et al. [17]	2018	54 mixed types	232	Face mask ventilation was conducted in 167 cases (72%); in three cases, it was inadequate. Intubation was used in 65 procedures and was difficult in 29% of them.
Cohen et al. [19]	2017	34 sanfilippo patients	86	Mask ventilation was easy in all patients. Endotracheal intubation was used in 73.2% of cases (63 occasions). Of those, two were seen difficult.
Kamata et al. [20]	2017	25 sanfilippo patients	43	No difficulties in mask ventilation during the procedures. Upper airway obstruction was seen in 14 procedures (33%) and was managed easily with continuous positive airway pressure, temporary oral airway placement, and jaw thrust. A small shoulder roll was required for 11 cases (26%). Tracheal intubation was not conducted.

Recent vs previous techniques

Despite all the problems associated with airway management in the MPS population, many cases have been secured successfully [1,21,22]. Using a supraglottic device to aid fiber-optic intubation can be very useful in MPS patients. Laryngeal mask airway, which is, a previous accessible device, has facilitated fiber-optic intubation in many patients adequately [11,12,15,17]. However, laryngeal mask airway should be used with caution in patients with high larynx and may be difficult to insert for patients who cannot open their mouths adequately [14,23]. I-gel is a recently presented supraglottic device that has been a perfect choice on many occasions [24-26]. I-gel has been reported as the best supraglottic device to facilitate intubation when it was compared with other supraglottic devices [26]. However, I-gel may not be appropriate for all mouth sizes [25]. Intubation using video laryngoscope has been in use only in recent years as a substitute intubation technique, and since it had been introduced, many MPS cases were intubated with no problems [15-19]. Tracheal intubation of MPS patients using video laryngoscope is superior to the use of either direct laryngoscopy or fiber-optic scope [15]. Direct laryngoscopy has been complicated even with many attempts [27]. However, video laryngoscope was not able to provide adequate vision in some cases, and fiber-optic scope was used instead [28]. Access to all the newly and previously introduced devices must be available to avoid any unexpected events. After more evidence regarding video laryngoscope superiority would be presented, the worldwide handiness of this newer and quicker technique will make it a preferred method in MPS patients who may no more develop complications regarding intubation placement. Another benefit of these recent techniques is decreasing the necessity for tracheostomy in MPS patients [11,12]. Avoiding tracheostomy in MPS is always a better choice due to the anatomical changes during the progression of the disease. However, the necessity of tracheostomy was mandatory in some cases [1]. A summary of the studies showing an overview of use of various techniques in MPS patients is shown in Table 3.

**Table 3:** Various Airway Management Techniques in Mucopolysaccharidoses Patients

Author Name/Year of Publication	Disease Type	Surgery/Age (years)	Clinical and Physical Features	Airway Securing
Hack et al. 2016 [1]	IH/S	C1–4 laminectomy and occipitocervical fusion (C0–C5) for cervical stenosis/27	Signs of cervical myelopathy, limited neck movement, severe micrognathia, severe obstructive sleep apnea, restrictive lung disease	Awake fiber-optic intubation was done. In the end, tongue and lip swelling was noted. The patient was extubated on postoperative day two.
Hack et al. 2016 [1]	IH/S	Mitral valve replacement/25	The patient had become breathless due to untreated severe mitral stenosis. Later, mitral regurgitation with pulmonary hypertension occurred.	Fiber-optic intubation was conducted successfully. After the surgery was done, the patient underwent a tracheostomy (to facilitate any prolonged period of ventilation if required). The patient called for five days of postoperative ventilator support. Weeks later, the patient made a good recovery.
Hack et al. 2016 [1]	II	ENT surgery/18	Hoarse voice, difficulty in breathing, and subsequent choking on eating. Severe obstructive sleep apnea with daytime somnolence. A CT scan of his thorax showed a twisting trachea with a narrowing in the subglottic and distal tracheal regions.	Awake fiber-optic intubation was done. Tracheostomy was conducted despite the anatomical difficulties. The patient was discharged home after two months.
Kadic et al. 2012 [21]	IV	Total hip replacement/31	Pain on the hips, deformity of the joints with bilateral coxarthrosis. The patient had a large head, short neck, large tongue, and abnormal shape of the spine on the radiograph	Awake fiber-optic intubation was conducted successfully, followed by awake extubation.
McLaughlin et al. 2010 [22]	IV	Total hip replacement/26	The patient had short stature, with a large head, short neck, macroglossia, enlarged uvula and tonsils. Kyphosis, anterior beaking of the cervical vertebrae on a radiograph and barrel chest were also noticed. The patient was diagnosed with osteoarthritis of the hips secondary to avascular necrosis.	Awake fiber-optic intubation was conducted. The trachea was extubated awake, and recovery was uncomplicated.
Michalek et al. 2008 [24]	II	Dental procedure under general anesthesia, which included radiographs, cleaning, polish, five fillings, and four extractions/25	Snoring at night, sleep apnea, dysphagia, gastric reflux with repeated aspiration cases of pneumonia, otitis media, multiple organs enlargement. The thyromental distance was 5.5 cm; neck mobility was extremely limited; and big tongue, and small mandible were also noted.	Mask ventilation was difficult, and a size-4 I-gel was inserted at the first attempt. Mechanical ventilation with an I-gel was effective in guiding the fiber-optic scope. General anesthesia lasted for 130 min. The postoperative period was uneventful, and the patient was dismissed the same day.
Dhanger et al. 2015 [25]	IV	Fixation of atlantoaxial subluxation/3	A weakness of lower extremities, coarse facies, macroglossia, large head, short neck, barrel chest, scoliosis, hyperreflexia, atlantoaxial subluxation, and rotation of C2 over C1	Fiber-optic bronchoscope was introduced through the I-gel into the trachea, and then the endotracheal tube was inserted, followed by removal of the bronchoscope and I-gel. Airway edema was noted, so the patient was moved to the intensive care unit. The child was extubated on the first postoperative day and discharged home on the eighth postoperative day.
Sayilgan et al. 2012 [27]	VI	Mitral valve replacement surgery/9	The patient suffered from dyspnea due to mitral valve prolapse and severe insufficiency. Physical examination showed microcephaly, coarse face, macroglossia, corneal opacity, gum hypertrophy, short neck, and kyphoscoliosis.	Intubation via direct laryngoscopy was unsuccessfully attempted four times. The patient was successfully intubated on the fifth attempt, and ventilation via mask was sufficient between intubation attempts.
Tsuchiya et al. 2019 [28]	(a) I, (b) II, (c) II, (d) I , (e) II, (f) VII	I) Scoliosis correction/28, all other patients had cervical decompression and their ages ranged between 13 and 38	Short neck with limited mobility, inability to fully open the mouth linked with macroglossia, abnormal enlargement of the epiglottis, arytenoid region, abnormal gate, limb weakness, compression of the spinal cord and disappearance of the normal curvature.	Face mask was difficult in two patients. Tracheal intubation was performed within 18 minutes in all six patients without a significant decrease in arterial oxygen saturation. Awake or under anesthesia fiber-optic intubation and video laryngoscope were used successfully. None exhibited airway edema or severe obstruction upon extubation.

Practice recommendations for airway management in MPS

Although the level of advantage for a particular technique cannot be specified from the literature, there is sufficient understanding between anesthesiologists that a preformed strategy can lead to better results. The aim of these recommendations (Table 4) is to prepare anesthesiologists to avoid airway-related problems in MPS patients. These recommendations that yield basic advice based on current published literature [3,6,29-31] can be revised and amended depending on clinical demands. However, it cannot warrant any particular consequence, and many patients may require additional assessment tools to figure out the nature of the predicted airway problem. Table 4 shows some recommendations for anesthesiologists for securing airway in MPS patients.

**Table 4:** Recommendations for Anesthesiologists

Preoperative Evaluation	Airway Securing Comments
All previously conducted anesthetic procedures must be reviewed. Physical airway examination and CT scan of the airway would be preferable. Radiographic evaluation to rule out any atlantoaxial subluxation. Assessment of sleep disorders and cardiorespiratory function is mandatory. At least, one additional individual must be available to help for unpredicted difficult airways.	Specialized equipment for airway management, including supraglottic devices and suitable tubes sizes, must be available. The correct position of the patient must be secured with stabilization of the neck. Consider under anesthesia or awake intubation. Confirm endotracheal tube placement. Intraoperative neurophysiologic assessment to predict spinal compression occurrence.

The impact of new MPS treatments on airway management

Recently, the quality of life in patients with lysosomal storage diseases, including MPS, has improved noticeably due to the remarkable development that has been reached in the field of therapy. Enzyme replacement therapy (ERT) and hematopoietic stem cell transplantation (HSCT) have been the standard of care in the MPS population. However, there is no satisfactory evidence regarding the impact of these treatments on reducing the anesthesiological risks. Two retrospective studies reported that treatment with ERT alone in MPS patients did not decrease the rate of difficult airways. At the same time, HSCT reduced the incidence of difficult mask ventilation and difficult endotracheal intubation when conducted at younger ages before the progression of the disease [11,32]. This can be due to the lack of a sufficient amount of ERT taken by younger patients since ERT needs to be administrated for a longer time.

On the other hand, a recent study showed no difference in airway management in patients who received HSCT and those who did not [18]. Also, HSCT has been associated with a high mortality rate [32]. ERT, followed by HSCT, could not lead to uneventful airway management in a retrospective report [15]. It has been evident that ERT has an insufficient influence on some vital organs such as bones, heart, and valves [33]. The inability of the ERT to cross the blood-brain barrier to curing the spinal cord compression and neurocognitive deterioration has made its impact more limited [3,33]. Significant restrictions regarding high mortality rate (HSCT), limited impact on vital organs (ERT), and the expenses issue have all together pushed toward finding more effective and better therapeutic methods. Further understanding of the pathophysiology of MPS has helped researchers to go beyond the points already reached regarding therapy agents. Some studies have offered enough proof that lysosomes are not there only to break down large molecules, but they can be engaged in many additional pathways such as signaling, adaptive immunity, plasma membrane trafficking, metabolism, and growth issues [34]. This broad involvement of lysosomes in several processes can contribute to difficult airways in MPS. Recently, some studies have investigated many therapeutic approaches, and some of them are promising. One approach was the recognition of all enzymes and processes involved in the formation of GAGs, which led to the development of substrate reduction therapy (SRT) [33]. SRT was able to decrease the level of GAGs deposits, improve the elasticity of connective tissues, and enhance the joints motion in MPS patients [35]. GAGs accumulation would no more be problematic whenever airways would be secured in MPS patients if enough studies would conclude the impacts of SRT. Unpredicted influence of GAGs deposits in MPS is the activation of the inflammation pathway, which can contribute to the pathological background to some of the disease manifestations such as skeletal abnormalities and nerve involvement [33]. Anti-tumor necrosis factor-alpha (anti-TNF α) has managed the musculoskeletal manifestations in MPS patients [33,36]. Some clinical trials have started evaluating the efficacy of adalimumab [36]. Anti-TNF α can lead to clinical improvement in MPS patients and interfere with the mechanism behind the joint and bone disease that was not managed adequately with ERT. In the future, giving anti-TNF α along with ERT in MPS patients can lead to better consequences, since ERT alone had a limited impact on some organs.

Pentosan polysulfate, an anti-inflammatory drug that has a pro-chondrogenic effect, was used in MPS I and could improve both pain and joint mobility [37]. More clinical studies in the future should be conducted to look for other additional benefits of pentosan polysulfate, compare it with anti-TNF α, and demonstrate whether its impact differs from one MPS type to another. Gene therapy, which can be a permanent therapy for MPS patients, is also promising and is currently under investigation by some studies that have not been completed yet [38,39].

When more studies in the future present enough evidence and explore additional impacts of these therapeutic agents, they can improve the quality of life in MPS patients a lot and make them less likely to undergo surgeries. Also, if they would be candidates for surgeries, these new therapies may reduce the rate of anesthesiologic complications in particular airway problems.

Limitations

There were certain limitations that we encountered in our review. The data included in this article was limited to humans only. Only those articles that were published in English language were selected; therefore, some valuable articles from other languages could have been missed. Randomized control trials and prospective studies of anesthesia management were lacking, and as the studies included were small populated, we could not find large samples. There is limited evidence regarding the efficacy of the new therapeutic agents in MPS patients, and several clinical trials of these treatments are not completed. In addition, more research should be conducted to explore its impact on airway management. 

## Conclusions

MPS patients require surgical procedures multiple times during the progression of the disease. However, the involvement of multiple organs and tissues leads to both clinical and physical problems regarding airway obtaining during surgical procedures. Access to recently introduced techniques and the availability of a team with advanced expertise in MPS diseases can reduce the incidence of airway-related complications dramatically. The impact of the previous MPS therapies on the airway is controversial. New treatment choices have been explored recently. However, their efficacy and exact impacts on different body organs and tissues are still under investigation.
